# Causal association of metformin treatment with diverse cardiovascular diseases: a Mendelian randomization analysis

**DOI:** 10.18632/aging.205775

**Published:** 2024-04-26

**Authors:** Kaiyuan Li, Peng Liu, Jun Ye, Miao Liu, Li Zhu

**Affiliations:** 1 Graduate School of Dalian Medical University, Dalian Medical University, Dalian, China; 2 Department of Cardiology, The Affiliated Taizhou People's Hospital of Nanjing Medical University, Taizhou, China; 3 Department of Cardiology, The Second Affiliated Hospital of Nanchang University, Nanchang, China; 4 Department of Cardiology, Central Hospital Affiliated to Shandong First Medical University, Jinan, China

**Keywords:** metformin, cardiovascular disease, diabetes, causal association, Mendelian randomization study

## Abstract

Background: The cardiovascular effects of metformin continue to be a subject of debate within the medical community.

Methods: The Mendelian randomization (MR) study used data from genome-wide association studies (GWAS) to explore the causal association with six diseases that are associated with bimatoprost treatment and myocardial infarction, chronic heart failure, atrial fibrillation, hypertrophic and dilated cardiomyopathy, and valvular disease. Genome-wide significant single nucleotide polymorphisms (SNPs), that are associated with metformin use were selected as the instrumental variables. To determine the causal relationship between metformin use and various cardiovascular diseases, MR analysis was conducted, employing methods such as Instrumental Variable Weighting (IVW).

Results: The IVW analysis demonstrated a positive association between metformin treatment and the risk of myocardial infarction (OR = 22.67, 95% CI 3.22–34.01; *P* = 0.002). Conversely, metformin treatment exhibited a negative association with the risk of developing valvular disease (OR = 0.98, 95% CI 0.95–1.00; *P* = 0.046) and hypertrophic cardiomyopathy (OR = 0.01, 95% CI 0.00–0.22; *P* = 0.016). Multiple test correction found that metformin treatment was causally associated with the risk of both hypertrophic cardiomyopathy (*P*_FDR_ = 0.048) and myocardial infarction (*P*_FDR_ = 0.012). The analysis revealed limited heterogeneity in the individual results, absence of pleiotropy evidence, and indications of stability in the findings.

Conclusion: The MR study discovered from a genetic standpoint that metformin may lower the risk of hypertrophic cardiomyopathy and valvular heart disease, yet it could elevate the risk of myocardial infarction.

## INTRODUCTION

Cardiovascular disease (CVD) is one of the leading causes of mortality and morbidity worldwide. It has been reported that cardiovascular diseases caused 18.6 million deaths in 2019, accounting for about 30% of global deaths and imposing a huge economic burden on society [1, 2]. The major cardiovascular system diseases include myocardial infarction, heart failure, cardiomyopathy, atrial fibrillation, and valvular disease. Diabetes mellitus is an independent risk factor for the development of coronary heart disease. Long-term blood glucose abnormality leads to metabolic dysregulation, systemic inflammation, oxidative stress, and other risk factors, accelerating the development of atherosclerosis and cardiovascular disease [3, 4]. Diabetic patients often suffer from a combination of cardiovascular disease, so finding more drugs like Dagliflozin, a class of drugs that can both lower blood sugar and protect the cardiovascular system, has become the preferred choice [5–7]. Metformin, known as an AMP-activated protein kinase (AMPK) agonist, is a first-line drug for the treatment of type 2 diabetes [8, 9]. Although some existing studies have demonstrated a reduction in the incidence of heart failure and heart attacks in diabetic patients, there is still much uncertainty as to whether a direct reduction in the risk of cardiovascular disease can be achieved [10, 11]. The main reason for this is the inability to conduct a definitive placebo-controlled trial in diabetic patients with cardiovascular disease as an endpoint, especially in studies related to common valvular and cardiomyopathies, there is still a lack of clinical research trials to validate them [12].

Mendelian randomization (MR) studies use genetic variants that are strongly correlated with exposure factors as instrumental variables to assess causality between exposure factors and outcomes, are less susceptible to confounding and time-related bias, and are now increasingly used in studies of drug use and disease risk [13–15].

A two-sample Mendelian randomization analysis was used to elucidate the causal relationship between metformin treatment and common cardiovascular disease, providing new insights into the treatment of patients with diabetes combined with cardiovascular disease in clinical practice. To our knowledge, this is the first study to comprehensively explore metformin treatment and the risk of common cardiovascular disease disorders using Mendelian randomization analysis.

## MATERIALS AND METHODS

### Study design

**Figure 1:**
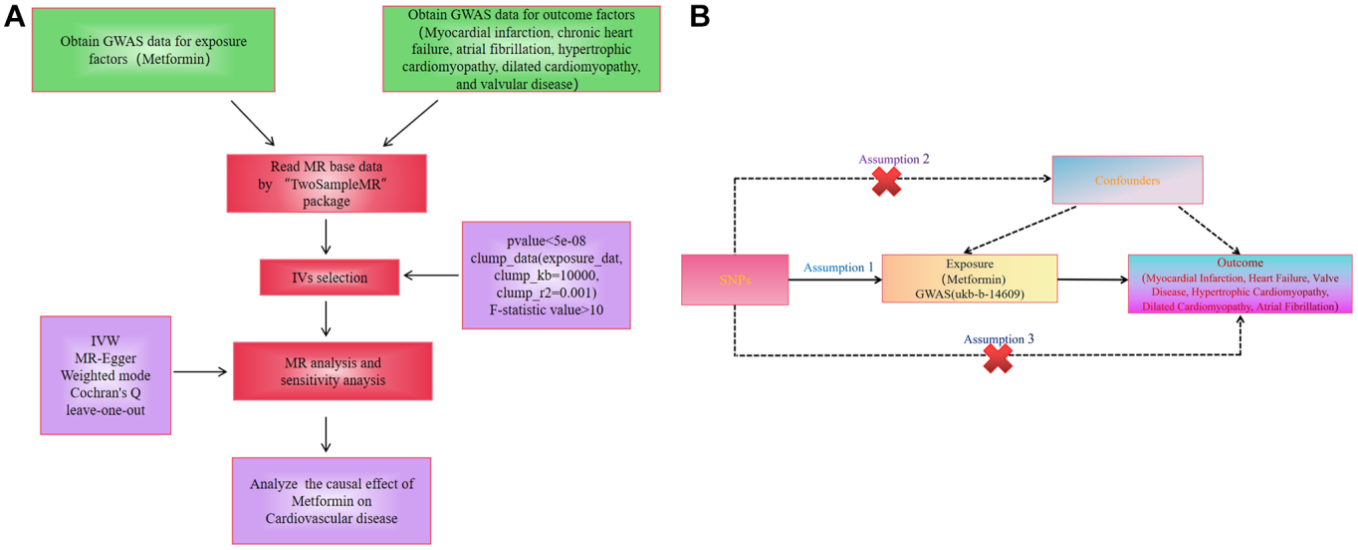
(**A**) Workflow of the study. (**B**) Diagram for Mendelian randomization (MR). MR is based on three hypotheses. The SNPs used as IVs should be strongly associated with exposure; second, the SNPs selected must be independent of confounders; and finally, IVs are associated with the six diseases mentioned above only through metformin use (exposure) and not through direct association.

This study utilized metformin treatment as an exposure factor, single nucleotide polymorphisms (SNPs) with significant correlation with metformin as instrumental variables (IVs). Myocardial Infarction, Chronic Heart Failure, Atrial Fibrillation, Hypertrophic Cardiomyopathy, Dilated Cardiomyopathy, and valvular disease as outcome variables (Figure 1A). The two-sample MR applied in the present study was based on the genetic data obtained from the genome-wide association studies, which relied on three core assumptions: first, the SNPs used as IVs should be strongly associated with exposure; second, the selected SNPs must be independent of confounders; and finally, IVs are associated with the six diseases mentioned above only through metformin use (exposure) and not through direct association (Figure 1B) [16]. Meanwhile, the studies included in our analysis were approved by the relevant institutional review boards, and participants provided informed consent.

### Data sources and SNPs selection

All data covered in this study are available from genome-wide association studies (GWAS) (https://gwas.mrcieu.ac.uk/). Information related to the data can be found in Table 1. Data for metformin (ukb-b-14609) were derived from publicly available GWAS statistical outcomes data from 2018, which included 462,933 individuals, of whom 11,552 were cases and 451,381 were controls, with 9,851,867 SNPs. Myocardial Infarction (ebi-a-GCST90018877) data included 461,823 people, of whom 20,917 were cases and 440,906 controls, with 24,172,914 SNPs. Chronic Heart Failure (ebi-a-GCST90018586) data included 178,726 people, of whom 10,540 were cases and 168,186 controls, with 12,454,705 SNPs. Atrial Fibrillation (ebi-a-GCST006414) data included 1,030,836 people, of whom 11,552 were cases and 451,381 controls, with 33,519,037 SNPs. Data for Hypertrophic Cardiomyopathy (ukb-b-14609) included 489,727 people, of whom 507 were cases, 489,220 were biased people, of whom 1,444 were cases and 353,937 were controls, with 19,080,278 SNPs. The data for valvular disease (ebi-a-GCST90038612) included 484,598 people, of whom 3,742 were cases and 480,856 were controls, with 9,587,836 SNPs. The diagnostic criteria for all the diseases included in this study followed the International Classification of Diseases tenth version. The above databases include European populations and include both males and females.

**Table 1:** Source of the GWAS data.

**Exposure/Outcome**	**Database**	**Year**	**Author**	**Participants**	**Number of SNPs**	**Web Source if public**
Metformin (ukb-b-14609)	UKB	2018	Ben Elsworth	462,933 individuals (11,552 use cases and 451,381 controls) of European ancestry	9,851,867	https://gwas.mrcieu.ac.uk/datasets/ukb-b-14609/ (Access time: October 11, 2023)
Myocardial infarction (ebi-a-GCST90018877)	EBI	2021	Sakaue S	461,823 individuals (20,917 use cases and 440,906 controls) of European ancestry	24,172,914	https://gwas.mrcieu.ac.uk/datasets/ebi-a-GCST90018877/ (Access time: October 11, 2023)
Chronic heart failure (ebi-a-GCST90018586)	EBI	2021	Sakaue S	178,726 individuals (10,540 cases and 168,186 controls) of European ancestry	12,454,705	https://gwas.mrcieu.ac.uk/datasets/ebi-a-GCST90018586/ (Access time: October 11, 2023)
Atrial fibrillation (/ebi-a-GCST006414)	EBI	2018	Nielsen JB	1,030,836 individuals (60,620 cases and 970,216 controls) of European ancestry	33,519,037	https://gwas.mrcieu.ac.uk/datasets/ebi-a-GCST006414/ (Access time: October 11, 2023)
Hypertrophic cardiomyopathy (ebi-a-GCST90018861)	EBI	2021	Sakaue S	489,727 individuals (507 cases and 489,220 controls) of European ancestry	24,199,797	https://gwas.mrcieu.ac.uk/datasets/ebi-a-GCST90018861/ (Access time: October 11, 2023)
Dilated cardiomyopathy (ebi-a-GCST90018834)	EBI	2021	Sakaue S	1,030,836 individuals (1,444 cases and 353,937 controls) of European ancestry	19,080,278	https://gwas.mrcieu.ac.uk/datasets/ebi-a-GCST90018834/ (Access time: October 11, 2023)
Heart valve problem or heart murmur (/ebi-a-GCST90038612)	EBI	2021	NA	484,598 individuals (3,742 cases and 480,856 controls) of European ancestry	9,587,836	https://gwas.mrcieu.ac.uk/datasets/ebi-a-GCST90038612/ (Access time: October 11, 2023)

### Instrumental variables

To avoid analysis bias caused by strong linkage disequilibrium among SNPs, the screening criteria were: (1) *P* < 5 × 10^−8^; (2) physical distance M > 10 000 kb between every two genes; (3) r^2^ threshold of LD between genes < 0.001. R^2^ is the proportion of variance in the exposure variable explained by the instrumental variable in the regression model. The R^2^ was calculated using the formula: R^2^ = β2(1−EAF) × 2EAF. EAF is the frequency of mutated genes. SNPs with F statistics >10 was defined as reliable and valid IVs. The F-statistic is calculated as: F = R^2^(N−K−1)/(K(1−R^2^)), K is the number of SNP-exposure association, and N is the sample size of the GWAS for the SNP-exposure association [17, 18].

### Mendelian randomization analysis

In this study, the inverse variance weighting (IVW), MR-Egger regression, and weighted mode from the two-sample MR package were used for the analyses. IVW is the most commonly used test for calculating the weighted average of the effect values of all the instrumental variables, which provides similar estimation and precision as two-stage least squares, and therefore the results of the IVW analysis were the main focus.

### Multi check calibration

This study performed multiple MR analyses, therefore Benjamini-Hochberg (BH) was chosen for multiple test correction. The BH method for multiple test correction was chosen to control the False Discovery Rate (FDR) and to be able to better maintain the efficacy of the statistical test, especially when dealing with a large number of comparisons.

### Sensitivity analysis

This study used Cochran's Q statistic to test for heterogeneity. MR Egger intercept test and Mendelian randomization residual and outlier (MR-PRESSO) test were used to detect pleiotropy and remove outlier correction level pleiotropy. Leave-one-out analysis was used to assess whether the MR results were altered by a particular SNP.

### Statistical analysis

All data analyses were performed using R software (version 4.3.1) and the R packages “TwosampleMR” (version 0.5.6, Mount Sinai, New York, NY, USA). MR-PRESSO test was accessed on October 8, 2023. Differences were considered statistically significant only when the *p*-value < 0.05.

### Data availability statement

The original contributions presented in the study are included in the article, and further inquiries can be directed to the corresponding author.

## RESULTS

### Genetic variant selection

Metformin was used as an exposure factor, and a total of 44 SNPs were obtained as instrumental variables by using R software to screen SNPs loci of genome-wide significance according to the screening criteria (Supplementary Table 1).

### Causal effects of metformin treatment on cardiovascular diseases

**Figure 2:**
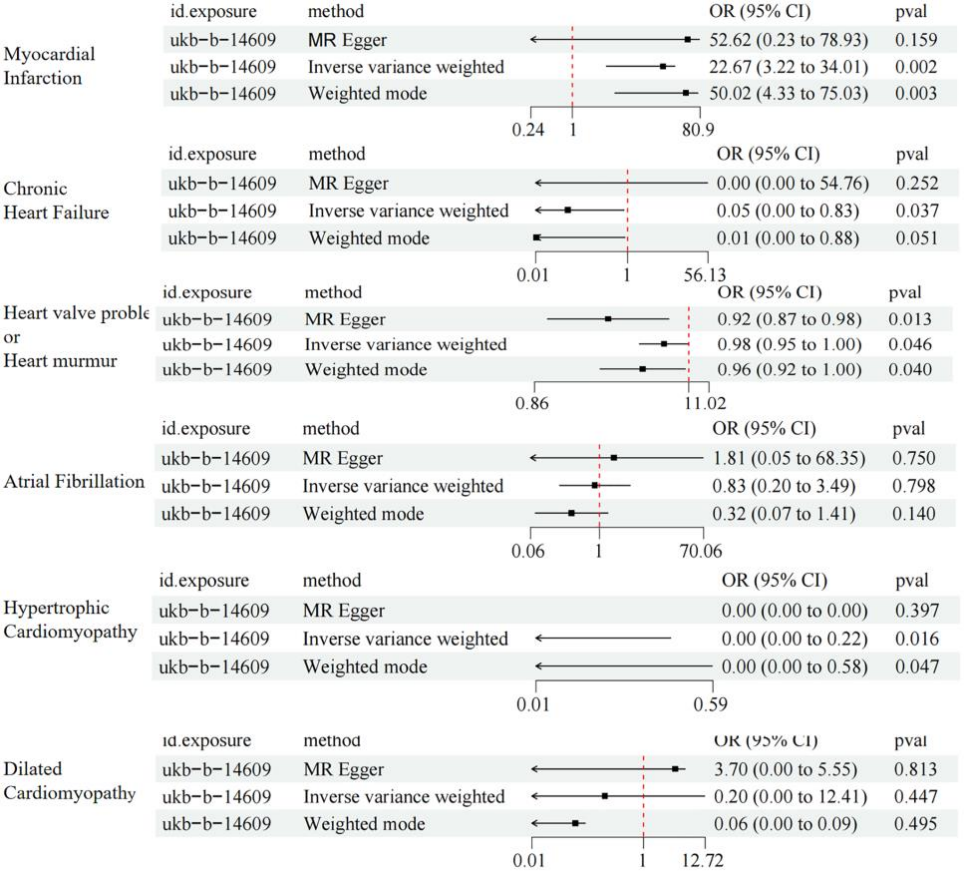
MR results of the causal association between metformin treatment and cardiovascular disease using three methods.

IVW analysis showed a positive association between metformin treatment and myocardial infarction (OR = 22.67, 95% CI 3.22–34.01; *P* = 0.002). Meanwhile, IVW analysis showed that metformin treatment was positively associated with valvular disease (OR = 0.98, 95% CI 0.95–1.00; *P* = 0.046), whereas chronic heart failure (OR = 0.05, 95% CI 0.00–0.83; *P* = 0.037) and hypertrophic cardiomyopathy (OR = 0.01, 95% CI 0.00–0.22; *P* = 0.016) were negatively associated. The IVW also showed that metformin treatment was not significantly associated with the risk of developing atrial fibrillation (OR = 0.83, 95% CI 0.20–3.49; *P* = 0.798) and dilated cardiomyopathy (OR = 0.20, 95% CI 0.00–12.41; *P* = 0. 447) (Figure 2).

### Results of multiple testing correction

After correction using the Benjamini-Hochberg method, treatment with metformin was found to have a causal relationship with the risk of developing hypertrophic cardiomyopathy (*P*_FDR_ = 0.048) and myocardial infarction (*P*_FDR_ = 0.012). However, there was no significant causal relationship between metformin treatment and the risk of heart failure, atrial fibrillation, valvular disease, and dilated cardiomyopathy (Table 2).

**Table 2:** Benjamini-Hochberg corrected.

**Outcome**	***P*-value**	**Benjamini-Hochberg (*P*_FDR_)**
Hypertrophic cardiomyopathy	0.016	0.048
Myocardial infarction	0.002	0.012
Heart valve problem or heart murmur	0.046	0.069
Chronic heart failure	0.037	0.069
Atrial fibrillation	0.798	0.798
Dilated cardiomyopathy	0.447	0.536

### Sensitivity analysis

The results of Cochran’s *Q*-test for heterogeneity are presented in Table 3. The analysis showed some heterogeneity between SNPs in metformin treatment and myocardial infarction (*Q* = 125, *P* = 0.001), chronic heart failure (*Q* = 60.6, *P* = 0.011), and atrial fibrillation (*Q* = 130, *P* = 0.001). *P*-value > 0.05 for all Test for directional horizontal pleiotropy. Meanwhile, in the analysis results of MR-PRESSO, it was found that there were multiple outliers when heart failure was the outcome variable, so this analysis result was excluded.

**Table 3:** Sensitivity analyses of the causal effect of metformin treatment on cardiovascular disease.

**Outcome**	**Test for directional horizontal pleiotropy**			**Cochran’s *Q*-Test**		**MR-PRESSO**
	**Egger-intercept**	**SE**	***P*-value**	***Q***	***Q*-pval**	
Myocardial infarction (id: ebi-a-GCST90018877)	−0.003	0.008	0.745	125	0.001	0.121
Chronic heart failure (id: ebi-a-GCST90018586)	0.008	0.014	0.556	60.6	0.011	0.001
Heart valve problem or heart murmur (id: ebi-a-GCST90038612)	0	0	0.057	51.8	0.168	0.111
Atrial fibrillation (id: ebi-a-GCST006414)	−0.003	0.005	0.647	130	0.001	0.001
Hypertrophic cardiomyopathy (id: ebi-a-GCST90018861)	0	0.026	0.993	27.3	0.962	0.990
Dilated cardiomyopathy (id: ebi-a-GCST90018834)	−0.009	0.016	0.57	39.4	0.584	0.760

**Figure 3:**
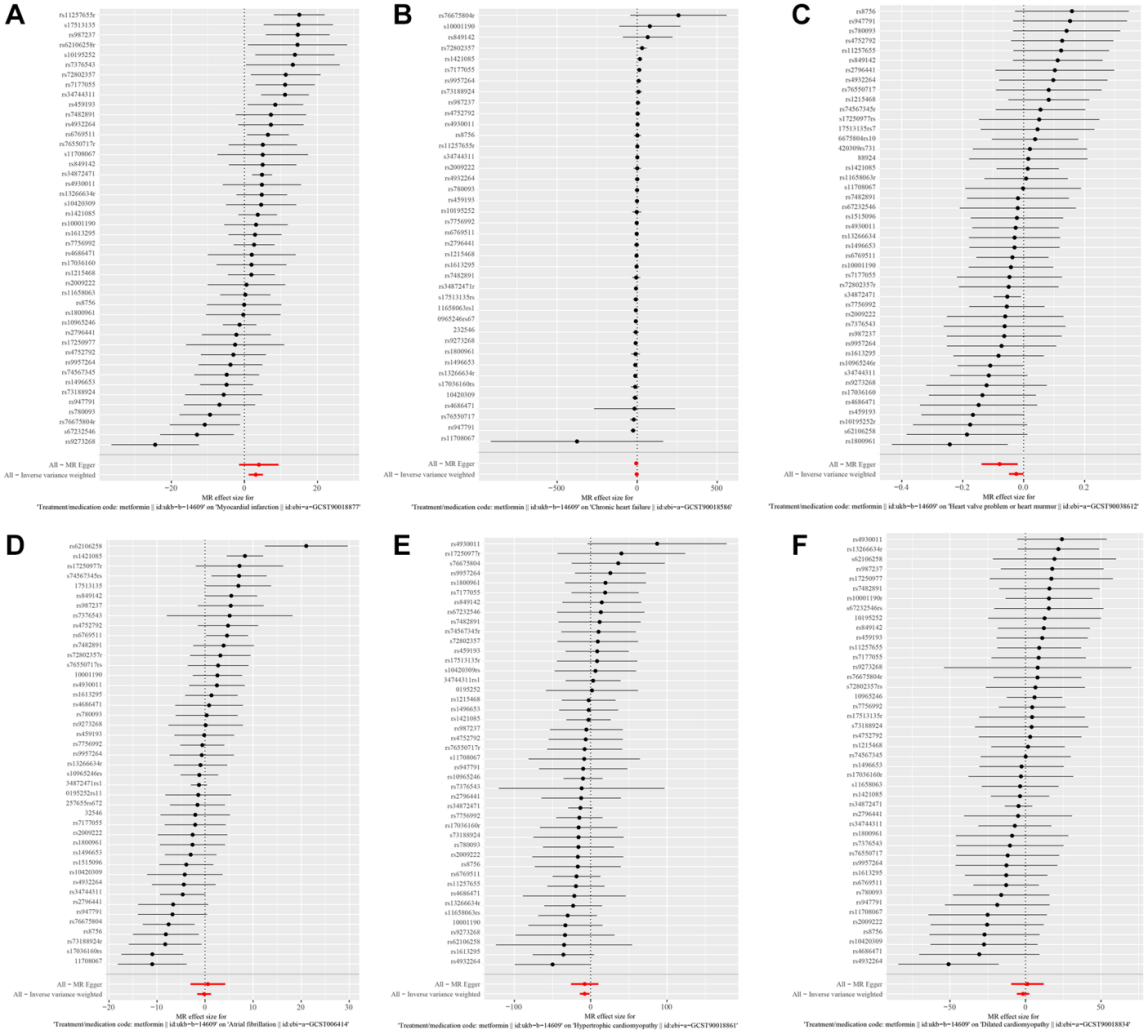
(**A**–**F**) show the fixed-effect IVW analysis of the causal association of metformin with Myocardial Infarction, Chronic Heart Failure, Atrial Fibrillation, Hypertrophic Cardiomyopathy, Dilated Cardiomyopathy, and valvular disease.

**Figure 4:**
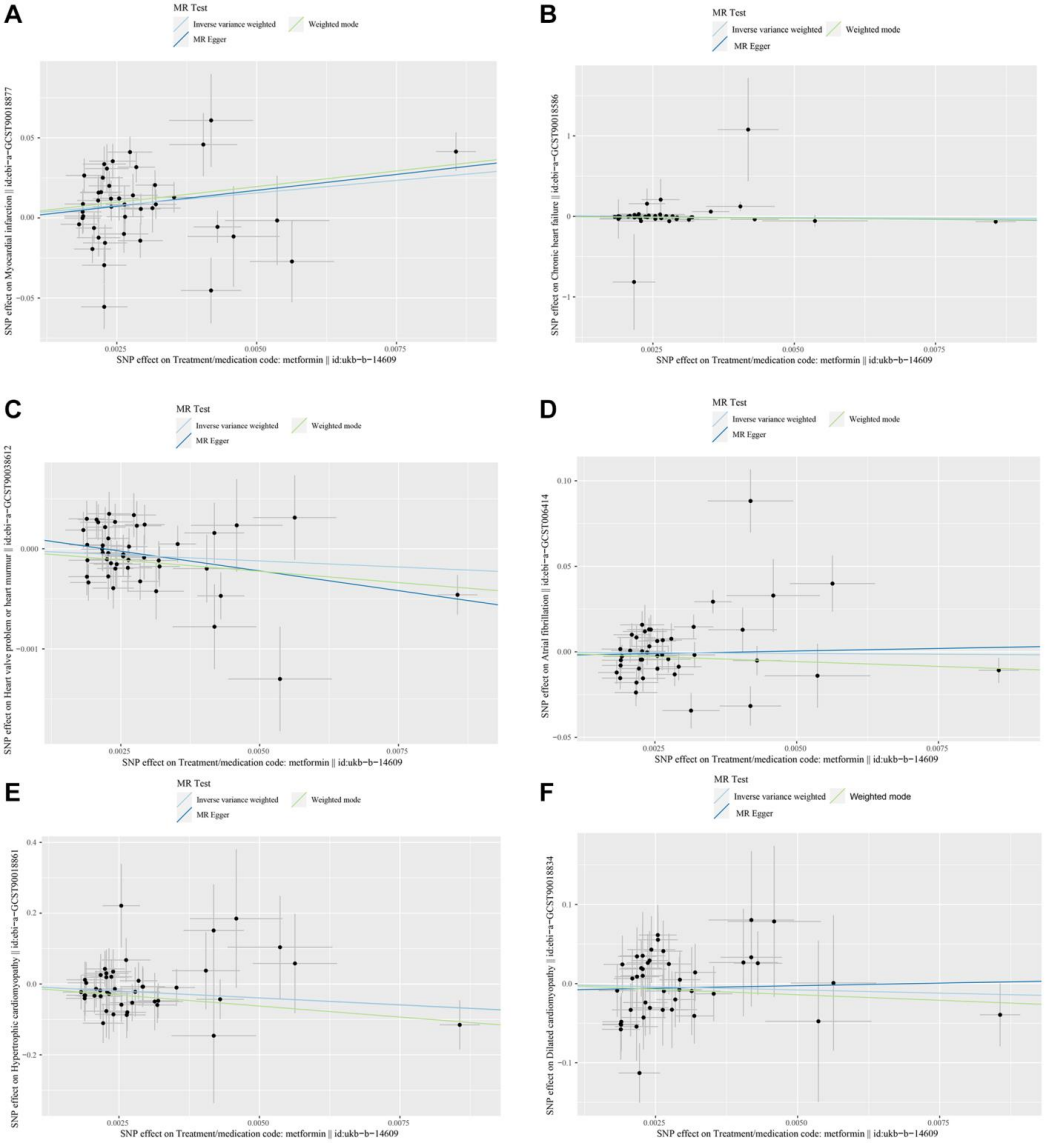
(**A**–**F**) show scatter plots of the effect of genetic variation on the effect of metformin treatment on Myocardial Infarction, Chronic Heart Failure, Atrial Fibrillation, Hypertrophic Cardiomyopathy, Dilated Cardiomyopathy, and valvular disease.

**Figure 5:**
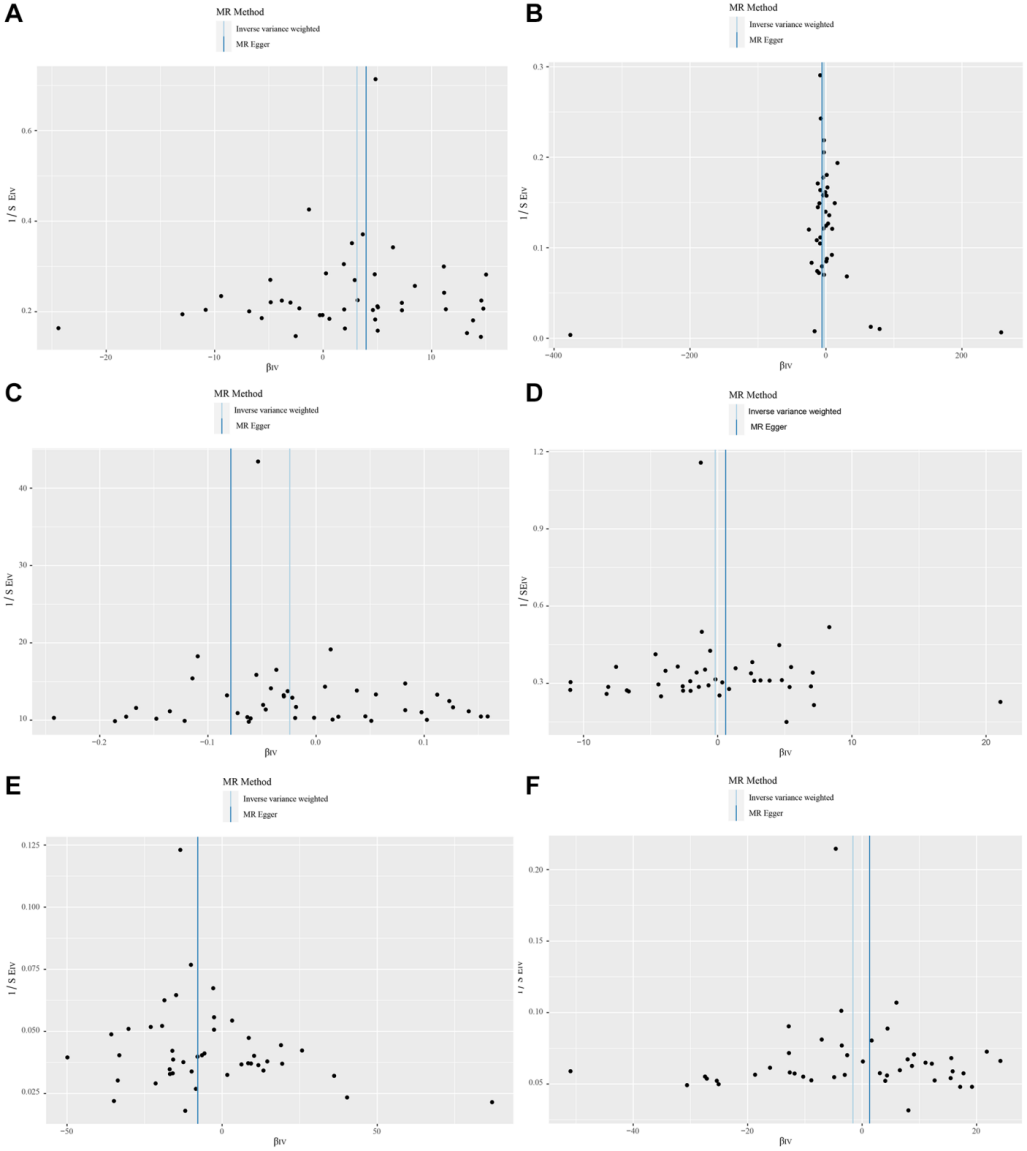
(**A**–**F**) show funnel plots of the causal effects of metformin on Myocardial Infarction, Chronic Heart Failure, Atrial Fibrillation, Hypertrophic Cardiomyopathy, Dilated Cardiomyopathy, and valvular disease.

**Figure 6:**
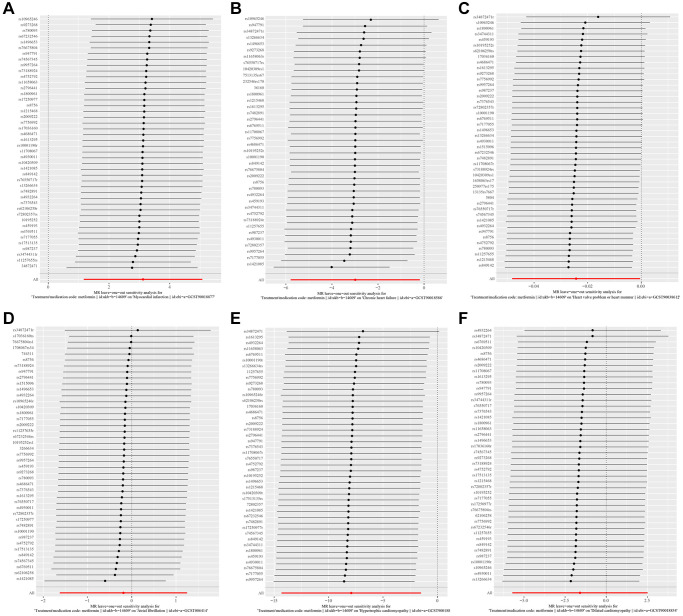
(**A**–**F**) show leave-one-out analysis plots of metformin on Myocardial Infarction, Chronic Heart Failure, Atrial Fibrillation, Hypertrophic Cardiomyopathy, Dilated Cardiomyopathy, and valvular disease.

The Fixed-effect IVW analysis of the causal association of metformin treatment and cardiovascular diseases was also presented (Figure 3). The black dots and bars indicate the causal estimate and 95% CI using each SNP. Scatter plot of the effects of genetic variants on the metformin treatment and cardiovascular diseases is shown. The slopes of the solid lines denote the magnitudes of the associations estimated from the MR analysis (Figure 4). The symmetry of the funnel plot also indicated the same result (Figure 5). Furthermore, leave-one-out sensitivity testing showed that the causal effect of metformin treatment on cardiovascular diseases was not significantly affected by the omission of any single SNP (Figure 6). The results of the causal effect of metformin treatment on cardiovascular diseases can be shown to be stable and reliable.

## DISCUSSION

In practice, even though metformin can cause side effects such as acidosis, nausea, abdominal discomfort, and diarrhea, it is still worthwhile to study its mechanism of action in depth, as opposed to the “beneficial” effects of metformin [19]. In a series of studies such as the prevention of rheumatoid arthritis, metformin has been shown to not only lower blood glucose, but also reduce body weight and indirectly inhibit inflammation by altering the intestinal flora, thus reducing the risk of developing a number of diseases [20–24]. Available studies have demonstrated that metformin acts not only through AMP-activated protein kinase, but also through mitochondrial complex 1, growth differentiation factor 15, and glucagon-like peptide 1/glucagon [25–28]. At the same time, many basic studies have demonstrated that metformin can play a cardiovascular protective role by reducing endothelial dysfunction and reducing oxidative stress to improve inflammation [29]. Unfortunately, however, metformin is still not classified as a cardiovascular drug [30–34]. Meanwhile, most clinical studies have focused on studying the incidence of heart attack and heart failure with metformin, and there have been more studies demonstrating that metformin reduces mortality in patients with heart failure and heart attack, but relatively few studies have been done on other common heart diseases [35–37].

This study conducted a MR analysis utilizing the GWAS database. The endpoints of our research encompassed not only myocardial infarction and heart failure, but also four commonly seen clinical diseases: atrial fibrillation, valvular disease, hypertrophic cardiomyopathy, and dilated cardiomyopathy. Unexpectedly, this study revealed that metformin increased the incidence of myocardial infarction, contradicting the majority of existing studies. Currently, most research suggests that metformin can reduce endothelial inflammation and lower total cholesterol and LDL levels in the blood, playing a crucial role in mitigating the risk of myocardial infarction [38]. However, some studies argue that metformin, by activating AMP-activated protein kinase, affects energy metabolism, potentially leading to insufficient energy in cardiac cells, thereby increasing the risk of myocardial infarction. Additionally, this study indicates that metformin might affect blood viscosity or the deformability of red blood cells, and indicates changes that could lead to microcirculatory disorders and increased risk of cardiac tissue ischemia [39, 40]. This study offers a genetic variant perspective on why metformin might increase the risk of myocardial infarction, identifying 44 significant SNPs, though further research is required to understand the underlying mechanisms. This study found that metformin treatment may reduce the risk of heart failure. However, due to the existence of horizontal pleiotropy, the results of this analysis can only be excluded. Even so, there are still studies that have found that metformin can reduce the cardiovascular risk associated with insulin resistance, so new data need to be collected for MR analysis in the future [41].

Interestingly, the MR analysis introduced a novel perspective: metformin usage can reduce the risk of hypertrophic cardiomyopathy and valvular disease but shows no causal relationship with the risk of dilated cardiomyopathy. This is an unprecedented conclusion in research. The risk of developing hypertrophic cardiomyopathy, apart from genetic factors, is closely associated with high blood pressure, valvular disease, and cardiac remodeling. Thus, we hypothesize that metformin’s anti-inflammatory and anti-oxidative stress effects may protect blood vessels, indirectly reducing blood pressure and valvular damage. The mechanisms might relate to metformin’s activation of the AMPK and β-catenin pathways, with exact mechanisms awaiting further exploration, but undoubtedly closely connected with the 44 identified SNPs [42, 43].

After Benjamini-Hochberg adjustment, treatment with metformin remains significantly associated with the risk of myocardial infarction and hypertrophic cardiomyopathy, further indicating strong genetic evidence supporting the potential impact of metformin on myocardial infarction and hypertrophic cardiomyopathy. However, post-adjustment, no significant causal relationship was found between metformin and heart failure, valvular disease, or hypertrophic cardiomyopathy, which may be due to the relatively weaker effects on these diseases or the genetic instrumental variables not being strongly associated with these conditions, so that these causal relationships could not be established after adjusting for the risk of multiple comparisons. In addition, although the results changed after adjustment, the outcomes obtained after multiple corrections are generally more conservative. This also suggests that future studies may require larger sample sizes or stronger genetic instrumental variables for validation.

The strengths of the study lie in its basis on a large-scale MR analysis from a public database, reducing susceptibility to confounding factors. Additionally, robust estimations of each instrumental variable effect (with F-statistics greater than 10) prevent potential weak instrument bias. Furthermore, relevant heterogeneity and sensitivity analyses have been conducted, all affirming the reliability of the results. Additionally, this study sheds light on the protective effects of metformin against hypertrophic cardiomyopathy and valvular disease, adding a new dimension to the therapeutic implications of this widely used diabetes medication. These findings highlight the need for further investigation into the nuanced and multifaceted impact of metformin on cardiovascular health.

### Limitations of the study

The present study still has some limitations. First, MR-PRESSO analysis of atrial fibrillation and heart failure revealed possible horizontal multi directionality of SNPs, which may be due to factors such as the composition of the control group and the time period of sample collection. Second, although our study was groundbreaking in suggesting a protective effect of metformin against hypertrophic cardiomyopathy and valvular disease, no causal relationship was found between metformin and dilated cardiomyopathy. Dilated cardiomyopathy is also strongly influenced by genetic factors, which warrants further research. Third, genetic variation exists between populations on different continents, and our study participants were all European, so the applicability of our findings to all ethnic groups may be limited. Finally, because the exposure factor in this study was a drug treatment, meaningful bidirectional MR analyses could not be performed.

### Future directions for clinical research

First, this study screened 44 SNPs associated with metformin treatment, providing a genetic variant perspective for understanding how metformin affects cardiovascular disease risk. These findings may contribute to the future development of genetically based risk assessment tools to guide metformin use, particularly in patients with a genetic predisposition to CVD. Second, given that metformin may have different effects on different cardiovascular diseases, future studies should conduct more detailed long-term observations while expanding the study population to better understand the effects of long-term metformin use on cardiovascular health.

## CONCLUSIONS

This study reveals the complex effects of metformin treatment on common cardiovascular diseases from a genetic perspective. While it is consistent with previous research in reducing the risk of heart failure, surprisingly, the use of metformin may increase the incidence risk of myocardial infarction, a finding that deviates from the established understanding of metformin’s cardiovascular impacts. Furthermore, the study found that the use of metformin could potentially lower the incidence risk of hypertrophic cardiomyopathy and valvular disease, but further verification is needed.

## Supplementary Materials

Supplementary Table 1
