# Supplementary material for: Causal association of metformin treatment with diverse cardiovascular diseases: a Mendelian randomization analysis
Source: Aging (Albany NY). 2024 Apr 26;16(9):7668–82. doi: 10.18632/aging.205775 (PMC11132001; doi:10.18632/aging.205775)
Supplement: Supplementary Table 1 [file aging-16-205775-s001.pdf]

SUPPLEMENTARY TABLE

Supplementary Table 1. The characteristics of SNPs and their genetic associations with metformin and cardiovascular disease.

SNP	SD	R2	F
rs17513135	0.262478827	7.51E-05	34.76479383
rs62106258	0.511951964	6.69E-05	30.9532236
rs1515096	0.242452174	0.000110077	50.96389706
rs10195252	0.224271425	7.30E-05	33.81789047
rs780093	0.225902324	8.34E-05	38.62203695
rs76675804	0.367084303	0.000129919	60.15124163
rs11708067	0.256680528	7.12E-05	32.94766208
rs17036160	0.342626259	8.38E-05	38.79153567
rs1496653	0.27314669	0.000114218	52.8812319
rs6769511	0.23680084	0.000180562	83.60269029
rs4686471	0.226642591	6.95E-05	32.16554861
rs10001190	0.228376909	0.000133339	61.73519549
rs7376543	0.264590763	7.70E-05	35.65491448
rs459193	0.252502922	8.76E-05	40.57668785
rs74567345	0.510535388	0.000121646	56.32073875
rs17250977	0.560322378	6.70E-05	31.02535276
rs9273268	0.281355617	6.53E-05	30.24787197
rs7756992	0.248743757	0.000165208	76.4924493
rs987237	0.286649065	7.17E-05	33.17300638
rs849142	0.21999108	0.000118609	54.91427422
rs13266634	0.238229663	0.000113923	52.74462392
rs2796441	0.222704483	6.72E-05	31.08989641
rs10965246	0.288978046	0.000221363	102.4984437
rs34872471	0.242369847	0.001246825	577.9144681
rs34744311	0.22747539	0.000156736	72.56942325
rs1613295	0.223005896	0.000115949	53.68247685
rs11257655	0.270950385	0.00010188	47.1682845
rs4752792	0.220698688	8.98E-05	41.58554008
rs67232546	0.270897314	7.05E-05	32.62157615
rs947791	0.267029287	7.35E-05	34.01578906
rs7482891	0.227379455	9.14E-05	42.30375764
rs4930011	0.226457524	0.000125825	58.25572105
rs76550717	0.3023736	8.50E-05	39.36548692
rs8756	0.220456468	7.33E-05	33.92337861
rs1215468	0.243457793	0.000144695	66.99346099
rs7177055	0.244168803	8.49E-05	39.28866867
rs4932264	0.24821033	7.98E-05	36.94925556

rs72802357	0.412502497	9.62E-05	44.54955654
rs1421085	0.224289796	0.000246742	114.2529394
rs11658063	0.226810647	0.000136224	63.07109823
rs9957264	0.296168427	7.87E-05	36.42313317
rs2009222	0.227842121	6.94E-05	32.13917398
rs10420309	0.222913363	7.22E-05	33.4368981
rs1800961	0.635128734	7.14E-05	33.04536264
rs73188924	0.265171137	6.72E-05	31.1091987
